# Prophylactic Effect of a Therapeutic Vaccine against TB Based on Fragments of *Mycobacterium tuberculosis*

**DOI:** 10.1371/journal.pone.0020404

**Published:** 2011-05-24

**Authors:** Cristina Vilaplana, Olga Gil, Neus Cáceres, Sergio Pinto, Jorge Díaz, Pere-Joan Cardona

**Affiliations:** 1 Unitat de Tuberculosi Experimental, Fundació Institut per a la Investigació en Ciències de la Salut Germans Trias i Pujol, Universitat Autònoma de Barcelona, Crta Badalona, Catalonia, Spain; 2 CIBER Enfermedades Respiratorias, Bunyola, Balears, Spain; University of Palermo, Italy

## Abstract

The prophylactic capacity of the RUTI® vaccine, based on fragmented cells of *Mycobacterium tuberculosis*, has been evaluated in respect to aerosol challenge with virulent bacilli. Subcutaneous vaccination significantly reduced viable bacterial counts in both lungs and spleens of C57Bl mice, when challenged 4 weeks after vaccination. RUTI® protected the spleen less than BCG. Following a 9 month vaccination-challenge interval, protection was observed for the lungs, but not for the spleen. Survival of infected guinea pigs was prolonged by vaccination given 5 weeks before challenge. Inoculations of RUTI® shortly after infection significantly reduced the viable bacterial counts in the lungs, when compared with infected control mice. Thus, vaccination by RUTI® has potential for both the prophylaxis and immunotherapy of tuberculosis.

## Introduction

Despite the major efforts undertaken to eradicate tuberculosis (TB), it remains a major health problem, with approximately 1.8 million deaths, 9 million incident cases and 13 million prevalent cases worldwide every year [1]. One of the key priorities for tuberculosis research involves focusing on therapeutic and preventive strategies [2]. Multidrug resistance and co-infection with HIV affect the dynamics of both the infection and the disease, therefore both these factors need to be taken into account when designing new strategies to combat TB [1].

In order to stop the spread of this infection, most recent research has focused on designing new prophylactic vaccine candidates with better safety and immunogenic profiles to either boost BCG (by ameliorating its immunogenic profile and prolonging its protection), or replace it [3]. As numerous different aspects of TB need to be covered in order to achieve its final eradication [3], the “perfect” approach would appear to involve a single polyfunctional vaccine candidate that is able to prevent the infection of healthy individuals whilst at the same preventing the reactivation and reinfection of latently infected people.

The aim of the present project was to assess the protective capacity of the RUTI® vaccine given either as prophylaxis, or soon after the infection, i.e. before the onset of the infection induced immune response.Primarily designed as a therapeutic agent to shorten the chemotherapy treatment of latent tuberculosis infection (LTBI), RUTI® is developed under good manufacturing practices (GMP) in Badalona (Catalonia, Spain), by Archivel Farma [4].

The objectives of the present study were 1.- to determine the prophylactic effects produced by this vaccine in mice: short and long term; 2.- the prophylactic effect in guinea pigs and 3.- the protective effect when given soon after infection in mice.

## Results

### Short-term prophylactic effect experiment

**Figure 1:**
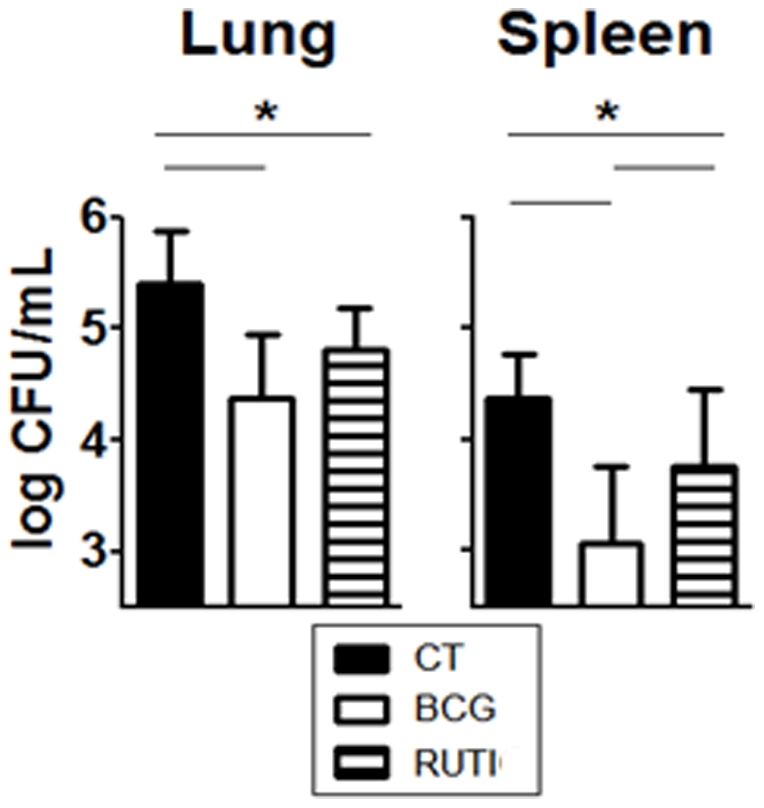
Protection after a short-term vaccination in terms of bacillary load obtained in tissues. The figure shows the reduction of the bacillary load in the lungs and spleen of animals according to their experimental group. The experimental groups are shown as follows: black (unvaccinated animals, control group), white (BCG vaccinated) and striped (RUTI® vaccinated). Significant differences (p<0.05) obtained after the statistical analysis (One-Way Anova) between groups are marked with an asterisk.

The effect of short-term vaccination on the bacillary load can be seen in Figure 1. RUTI® vaccine reduced the bacillary load in both lungs (0.58 log) and spleen (0.6 log) of mice, showing a statistically significant protective effect when compared to the control group (One-Way Anova, p<0.005). BCG appeared to be more effective than RUTI® (reduction in lungs: 1.04 logs; and in the spleen: 1.29), although the difference was only statistically significant in spleen (One-Way Anova, p<0.005).

### Long-term vaccination prophylactic effect experiment

**Figure 2:**
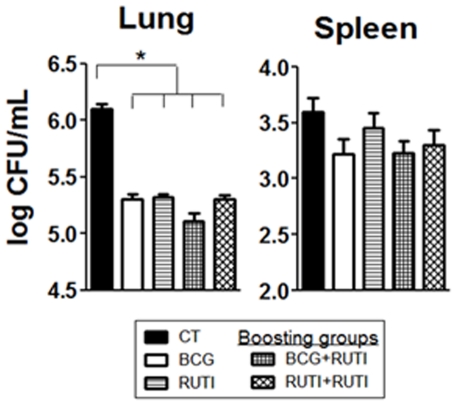
Results of the long-term vaccination experiment. The experimental groups are shown as follows: black (unvaccinated animals, control group), white (BCG vaccinated), striped (RUTI® vaccinated), chequered (BCG boosted with RUTI®) and with diamonds (RUTI® boosted with RUTI®). Significant differences (p<0.05) between groups are marked with an asterisk.

The results of the long-term vaccination experiment (Figure 2) show that every vaccination regimen had a protective effect in lung of mice. The decrease of the bacillary load in lungs was 0.78 and 0.8 logs for RUTI® and BCG respectively. This effect was statistically significant when compared to the control group (One-Way Anova, p<0.005). In spleen the reduction was lower: 0,38 logs for RUTI® and 0.15 for BCG. No differences were encountered between either the prophylactic regimens or the boosting ones, although animals vaccinated with BCG and boosted with RUTI® seemed to have better results than the other groups, at least in lung (reduction of 0.99 logs), with the difference being statistically significantly in one of the two repetitions of the experiment. As all animals were challenged nine months after the first vaccination, the long-lasting protective effect in lung achieved by both RUTI® and BCG is quite remarkable.

### Short-term prophylactic effect in guinea pigs

**Figure 3:**
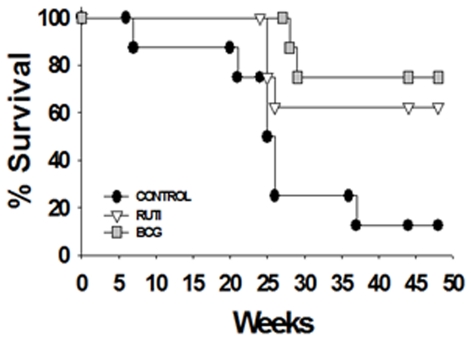
Survival experiment in guinea pigs after short-term vaccination. Data show the evolution of the protection given by the vaccination. Only the BCG group demonstrated a significant difference when compared with the control under the Kaplan-Meier Survival Analysis.

The results obtained in the survival experiment, which was ended at week 47 post-challenge, are presented in Figure 3. The Kaplan-Meier Survival Analysis shows a notable increase in the survival of the vaccinated animals, although only the BCG vaccination showed a statistically significant difference with respect to the control group.

### Protective effect when vaccinating soon after the infection

**Figure 4:**
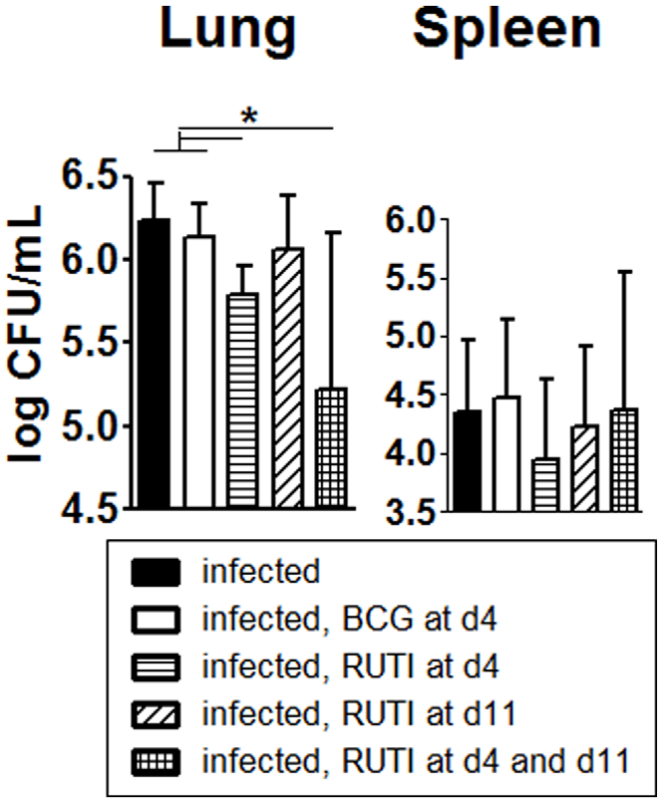
Vaccination soon after infection. The figure shows the protection induced by vaccinating after challenge. The experimental groups are shown as follows: black (unvaccinated animals, control group), white (BCG day 4), horizontal striped (RUTI® day 4), diagonal striped (RUTI® day 11) and chequered (RUTI® day 4 and 11). Significant differences (p<0.05) respect to the control group are marked with *.

The results (Figure 4) showed that RUTI decreased the bacillary load in mice lungs when given at day 4 (reduction of 0.53 logs) or at day 4 plus 11 (reduction of 1.03 logs) post challenge in a statistically significant way when compared to the infected and the BCG-vaccinated groups (One-Way Anova, p<0.005). No statistically significant differences where encountered in the spleen.

## Discussion

Vaccination is known to be the most cost-effective intervention to control TB, as explained elsewhere [5]. Indeed, the BCG vaccine has proven to be a useful tool over many years and has led to a reduction of the incidence of severe cases in infants. However, this vaccine seems to have poor efficacy especially in non-endemic countries, and systematic vaccination has been progressively abandoned [6], although it is still needed in areas with a high-prevalence of TB. Coinfection with HIV worsens the panorama as HIV-positive children vaccinated with BCG are at risk of developing a serious complication known as BCG disease [7], [8], [9]. As those countries with the highest rates of tuberculosis infection also have high rates of HIV infection, this is a serious cause for concern [8]. Furthermore, the increasing incidence of multidrug-resistant TB strains is also worrying. As a result, and in order to develop a better vaccine, the last few years have been very productive in terms of design of new vaccine candidates against TB. Indeed, some of the 12 new vaccine candidates already undergoing clinical trials have been designed as prophylactic vaccines in order to replace BCG or to boost it, whereas others are intended for use as therapeutic agents to accelerate or ameliorate chemotherapy, [10], [11]. These candidates are normally grouped on the basis of their nature (recombinant live, viral vectored, recombinant protein and other), although they can also be grouped on the basis of the vaccination strategy in which they were designed to be used (prime-boost, post-exposure, immunotherapy) [3]. It is well-known that TB is a difficult problem to approach as the clinical aspects of infection and disease are different and the immunopathogenesis of each one is as-yet unclear. Infected people also represent a big problem, both at a public health level as a reservoir of disease and at an individual level as 10% are susceptible to developing active TB. The “ideal” vaccine candidate would therefore be one which is able to prevent infection in a healthy person, trigger an immune response that is able to clear the infection in a recently exposed person, and to halt infection by decreasing the bacillary burden, thereby preventing reinfection and progress towards active disease, in a latently infected person. The best approach may therefore be to explore all the possibilities of these new candidates, as suggested recently [3] and has already been partially done in cases such as MVA85A, which has been evaluated in many different schedules and populations [12].

RUTI® is a vaccine candidate which was primarily designed to be included as an adjunctive to chemotherapy in an LTBI therapeutic regimen in order to reduce its duration and improve its efficacy. RUTI® is based on fragmented cells of *M. tuberculosis*, with the product being pasteurized, lyophilized and liposomed to allow better antigen presentation [4], and has demonstrated its immunogenicity against antigens traditionally related to the stationary phase of the bacilli (Rv 2031c, Rv 0934) [13]. The safety and efficacy of this vaccine have been tested in numerous experiments conducted in mice, guinea pigs, goats and minipigs, and the vaccine finally entered clinical development in 2007 [14], [15], [16], [17]. The phase 1 clinical trial (CT) proved it to be safe and immunogenic, thus allowing RUTI® to be evaluated in the context of a phase 2 CT, which began in South Africa at the second half of 2010 [18].

In spite of the murine models being so different than humans, mice were primary chosen as the best first screening to evaluate the impact of the prophylaxis administration of RUTI®. At short-term, both immunizations showed a statistically significant decrease on the bacillary load of more than half a log (both in lungs and spleen), less than the values usually reached using other candidates [19], BCG achieving better results than RUTI®. The effect of both immunizations on the bacillary load of lungs remained and was even higher than at short term in the case of RUTI® when challenging the mice 9 months after. In this long-term experiment RUTI® proved to be as good as BCG. Adding a boosting with RUTI® to BCG tended to increase this effect. As expected from evidences obtained in previous studies using RUTI® as a therapeutic agent, the boost with this vaccine candidate did not provoke a necrotic Koch reaction [20], [21].

Guinea-pigs were used in order to evaluate the impact of vaccination on the animals' survival, for their major susceptibility to *M.tuberculosis* infection, as has been done in other studies [22]. In this model, both RUTI® and BCG resulted in an increased survival when compared to the unvaccinated control, although the difference was only statistically significant for the BCG group, something that we relate to the low number of animals included in the study.

The results of RUTI® in the post-challenge experiments suggest that it could be given to recently infected people, in particular in those negative tuberculin skin test contacts of a tuberculosis index case. This would be feasible as the normal policy (in some countries like Spain) in these subjects is to provide chemoprophylaxis until retesting them with a tuberculin skin test (TST) two months later. If the TST remains negative, the chemotherapy is stopped, while being extended (up to 9 months) if the TST converts and the infection is confirmed [23]. The fact that protection is only achieved when RUTI® is administered at day 4 but not at day 11, suggests that it should be given as soon as possible after the exposure. If not, the immune response is not elicited quick enough to stop the bacillary load (i.e. in the case of being administered only at day 11). Besides, a second dose at day 11 achieves better results, and we assume this is because of boosting the immune response induced by the dose at day 4.

We believe that several conclusions can be extracted from our results. Thus, they reinforce the advantages of the therapeutic use of RUTI® against LTBI by adding a prophylactic effect against future reinfections, which is a major concern when trying to implement LTBI treatment in countries with a high risk of *M. tuberculosis* infection. They also include a new potential use on those persons with strong evidence of recent infection that have not developed yet any specific immune response. Data demonstrate a prophylactic effect of RUTI® which is long-lasting, thus suggesting that it should be tested as a candidate BCG booster. Further experiments will however be needed to determine the exact mechanism of action of this vaccine candidate, to explore its value in more situations and to finally establish its indications.

## Materials and Methods

**Figure 5:**
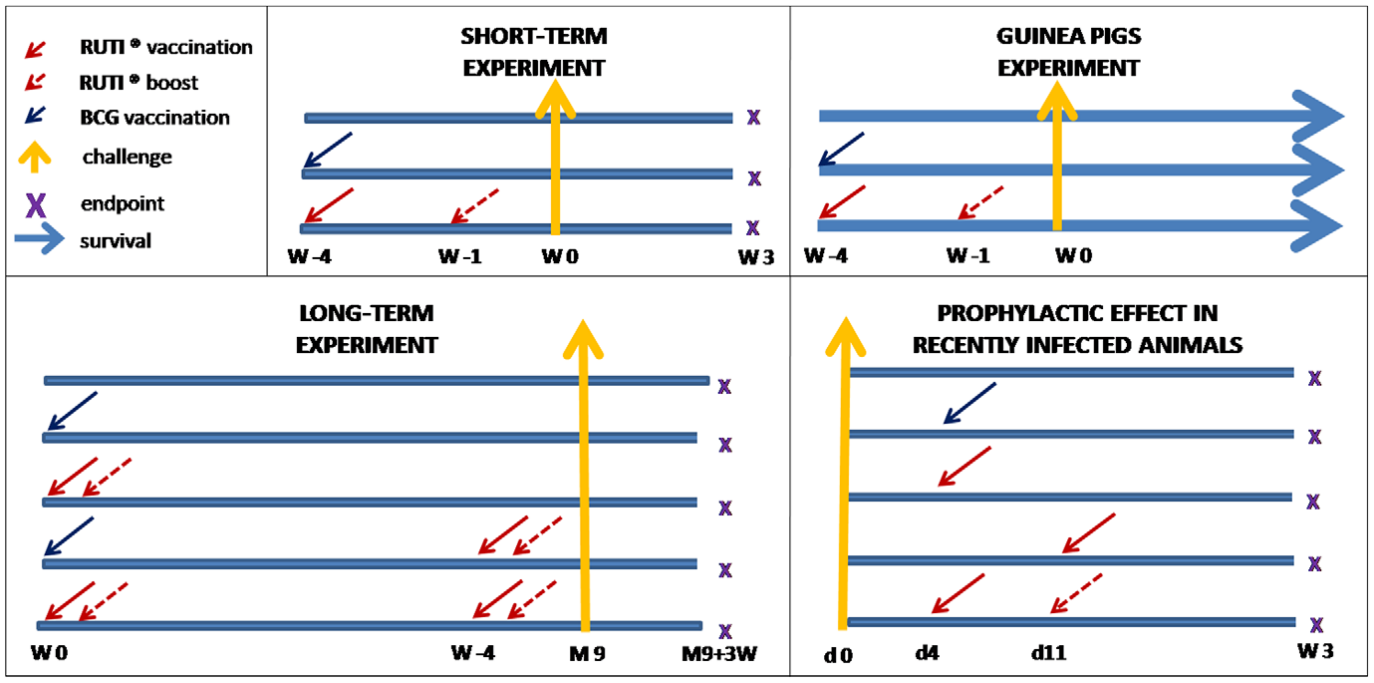
Experimental design of the experiments. The figure shows in 4 panels the experimental design of the 4 experiments run. As indicated in the figure, red arrows mean RUTI® vaccination (dotted if boosting) and the blue arrow means BCG vaccination. The purple X represents endpoint (mice sacrifice), the yellow vertical arrow the aerosol challenge and the blue horizontal arrow following-up to evaluate survival.

Different experiments were designed to evaluate any short- or long-term prophylactic effects of the vaccine in comparison with those triggered by BCG and to determine its effect on survival. One experiment was design to evaluate the effect of RUTI® when administered soon after challenge. The experimental design of the 4 experiments run is presented as Figure 5.

### Vaccination

RUTI® is based on detoxified fragments from *M. tuberculosis* cultured under stress conditions and liposomed, manufactured by Archivel Farma (Badalona, Catalonia, Spain) under GMP quality standards [14]. Vaccination with RUTI® (batch B06, 260 µg) was performed subcutaneously with two doses three weeks apart, as it is done when administered as a therapeutic approach. BCG vaccinated mice received a single dose of BCG Danish (SSI, Copenhagen, Denmark; 10^6^ CFUs), injected subcutaneously.

### Infection

*M. tuberculosis* strain H37Rv Pasteur was grown in Proskauer Beck medium. Animals were placed in the exposure chamber of an airborne infection apparatus (Glas-col Inc., Terre Haute, IN, USA) for infection with a low dose of *M. tuberculosis*. Nebulization provided an approximate uptake of 20–50 bacilli by mice lungs and 10 bacilli by guinea pig lungs.

### Ethics

All animal procedures were approved and supervised by the Animal Care Committee of the Germans Trias i Pujol University Hospital and by the Department of Environment of the Catalan Government (approval numbers 4092, 4095 and 4122). Mice and guinea pigs were weighed and checked every day following a protocol that monitored weight loss, apparent good health (bristled hair and wounded skin) and behaviour (signs of aggressiveness or isolation). Mice were euthanized with isoflurane and guinea pigs with ketamine (100 mg/kg) plus diazepam (5 mg/kg).

### Short-term prophylactic vaccination experiment. Experimental design

6–7-week-old female C57BL mice (Harlan Iberica, Sant Feliu de Codines, Catalonia, Spain) were used for a standard experiment [19] (performed in duplicate) involving the aerosol challenge of animals shortly after vaccination. Three experimental groups were established: a) infected non-vaccinated control; b) BCG vaccinated animals, and c) RUTI® vaccinated mice. Each group included 12 animals.

BCG and RUTI® vaccines were administered at week −4. A boost of RUTI® was given at week −1.

The mice were sacrificed three weeks after infection and lung and spleen samples extracted in order to evaluate the bacillary load.

### Long-term vaccination experiment. Experimental design

6–7-week-old female C57BL mice (Harlan Iberica, Sant Feliu de Codines, Catalonia, Spain) were used for this experiment (performed in duplicate). Five experimental groups were established : a) non-vaccinated control; b) BCG vaccinated animals; c) RUTI® vaccinated mice; plus two booster groups involving: c) BCG plus boosting with RUTI® and d) RUTI® plus boosting with RUTI®. Each group included 12 animals.

One single dose of BCG vaccine and two of RUTI® three weeks apart were given. Boosting with two doses of RUTI® was done in boosted groups at both four and one weeks before challenge. All animals were challenged by aerosol nine months after the first vaccination. Mice were sacrificed three weeks post-infection and lung and spleen samples extracted in order to evaluate the bacillary load.

### Short-term vaccination in guinea pigs. Experimental design

250-g specific pathogen-free female Dunkin-Hartley guinea pigs (Harlan Iberica, Sant Feliu de Codines, Catalonia, Spain) were used for this experiment. Three experimental groups were established (six guinea pigs per group): a) non-vaccinated control; b) single dose of BCG vaccine; c) two doses of RUTI® vaccine three weeks apart. Animals were challenged by aerosol 5 weeks after vaccination. The animals were then followed-up in order to establish the effect of the vaccination on their survival. Any animal which appeared to suffering or which suffered a 20% weight loss was sacrificed in order to comply with all ethical requirements. A Kaplan-Meier Survival Analysis was performed to detect any differences between the experimental groups and determine their statistical significance.

### Protective effect in recently infected animals. Experimental design

6–7-week-old female C57BL mice (Harlan Iberica, Sant Feliu de Codines, Catalonia, Spain) were used for this experiment (performed in duplicate). Animals were vaccinated shortly after challenge (day 0): with BCG on day 4, or with RUTI on day 4, 11 or 4 and 11. The mice were sacrificed three weeks after infection and lung and spleen samples extracted in order to evaluate the bacillary load.

### Evaluation of the bacillary load

Lungs and spleens of the animals were removed every planned timepoint, and mechanically disrupted in order to obtain tissue homogenates to be plated in triplicate on Becton Dickinson 7H11 Middlebrook agar (Bennex Ltd, Shannon, Ireland) and incubated at 37°C. Viable bacteria (Colony Forming Units, CFUs) were counted four weeks after, data being recorded as the log of the mean number of CFUs recovered per organ. The results of the different groups were compared and evaluated for statistically significant differences (One Way Anova test), with the differences being considered significant at *P*≤0.05.
